# The landscape of dementia research, diagnosis, treatment, and care in Latin America

**DOI:** 10.1002/alz.71309

**Published:** 2026-03-27

**Authors:** Claudia K. Suemoto, Nilton Custodio, Diego Aguilar, José A. Avila‐Funes, Sandra Baez, Pablo M. Bagnati, Lisa L. Barnes, Sonia M. D. Brucki, Ismael L. Calandri, Paulo Caramelli, Mario Cornejo‐Olivas, Carolina Delgado Derio, Sergio T. Ferreira, Adolfo M. García, Lea T. Grinberg, Agustin M. Ibanez, Rosario Isasi, Sylvia E. Josephy‐Hernández, Maria Lazo Porras, Jorge J. Llibre‐Guerra, Juan J. Llibre‐Rodriguez, Mychael V. Lourenco, B. Marcela Mar Meza, Joaquin Migeot, J. Jaime Miranda, Claudia Miranda‐Castillo, Catherine J. Mummery, José F. Parodi, Natalia Pozo Castro, Rosa María Salinas Contreras, Hernando Santamaría‐García, Andrea Slachevsky, Juliana N. Souza‐Talarico, Ezequiel I. Surace, Leonel T. Takada, Elena Tsoy, Martha Unaucho Pilalumbo, Eduardo R. Zimmer, Igor C Fontana, Simin Mahinrad, Heather M. Snyder, Maria C. Carrillo

**Affiliations:** ^1^ Division of Geriatrics University of Sao Paulo Medical School São Paulo Brazil; ^2^ Unidad de Investigación de Deterioro Cognitivo y Prevención de Demencia Instituto Peruano de Neurociencias Lima Peru; ^3^ Alzheimer's Disease International London UK; ^4^ Dirección de Enseñanza Instituto Nacional de Ciencias Médicas y Nutrición Salvador Zubirán Mexico City Mexico; ^5^ Univ. Bordeaux Inserm Bordeaux Population Health Research Center Bordeaux France; ^6^ Departamento de Psicología Universidad de los Andes Bogotá Colombia; ^7^ Department of Cognitive Neurology Neuropsychiatry and Neuropsychology Fleni Neurological Research Institute Buenos Aires Argentina; ^8^ Rush Alzheimer's Disease Center Rush University Medical Center Chicago Illinois USA; ^9^ Cognitive and Behavioral Neurology Unit, Faculdade de Medicina Universidade de Sao Paulo Sao Paulo Brazil; ^10^ Department of Cognitive Neurology Fleni Buenos Aires Argentina; ^11^ Department of Neurology Amsterdam Neuroscience Alzheimer Center Amsterdam Vrije Universiteit Amsterdam, Amsterdam UMC Amsterdam The Netherlands; ^12^ Behavioral and Cognitive Neurology Unit Faculdade de Medicina Universidade Federal de Minas Gerais Belo Horizonte Brazil; ^13^ Neurogenetics Working Group Universidad Cientifica del Sur Lima Peru; ^14^ Neurogenetics Research Center Instituto Nacional de Ciencias Neurologicas Lima Peru; ^15^ Departamento de Neurología y neurocirugía Unidad Cerebro saludable Hospital Clínico Universidad de Chile Santiago Chile; ^16^ Departamento de Neurociencias Facultad de medicina Universidad de Chile Santiago Chile; ^17^ Institute of Biophysics Carlos Chagas Filho Federal University of Rio de Janeiro Rio de Janeiro Brazil; ^18^ Institute of Medical Biochemistry Leopoldo de Meis Federal University of Rio de Janeiro Rio de Janeiro Brazil; ^19^ D'Or Institute for Research and Education Rio de Janeiro Brazil; ^20^ Cognitive Neuroscience Center (CNC) Universidad de San Andrés Buenos Aires Argentina; ^21^ Global Brain Health Institute (GBHI) University of California San Francisco California USA; ^22^ GBHI Trinity College Dublin Dublin Ireland; ^23^ Departamento de Lingüística y Literatura Facultad de Humanidades Universidad de Santiago de Chile Santiago Chile; ^24^ Mayo Clinic Jacksonville Florida USA; ^25^ Latin American Brain Health Institute (BrainLat) Universidad Adolfo Ibáñez Santiago Chile; ^26^ Department of Biophysics School of Medicine Istanbul Medipol University Istanbul Türkiye; ^27^ Barcelonaβeta Brain Research Center (BBRC) Pasqual Maragall Foundation Barcelona Spain; ^28^ Hussman Institute for Human Genomics Institute of Bioethics and Health Policy University of Miami Leonard M. Miller School of Medicine Miami Florida USA; ^29^ Hospital México Caja Costarricense de Seguro Social San José Costa Rica; ^30^ Universidad de Costa Rica San José Costa Rica; ^31^ CRONICAS Centre of Excellence in Chronic Diseases Universidad Peruana Cayetano Heredia Lima Peru; ^32^ Department of Neurology Washington University School of Medicine in St.Louis St.Louis Missouri USA; ^33^ Dementia Research Unit, Medical University of Havana Havana Cuba; ^34^ Programa M‐MENTE. Hospital Central Fuerza Aerea del Perú Lima Perú; ^35^ Coordinadora curso psico‐geriatría. Maestría en Geriatría y Gerontología Universidad Peruana Cayetano Heredia Lima Perú; ^36^ Sydney School of Public Health Faculty of Medicine and Health The University of Sydney NSW Australia; ^37^ Instituto de Investigación en Ciencias de la Salud Faculty of Nursing Universidad Andres Bello Santiago Chile; ^38^ Millennium Institute for Care Research Santiago Chile; ^39^ Dementia Research Centre, National Hospital for Neurology and Neurosurgery Institute of Neurology University College London London UK; ^40^ Universidad de San Martín de Porres Facultad de Medicina Centro de Investigación del Envejecimiento Lima Perú; ^41^ Neurology and Neurosurgery Department School of Medicine University of Chile Santiago Chile; ^42^ Neurology and Neurosurgery Department Hospital Clínico San Borja Arriarán Santiago Chile; ^43^ National Institute of Neurology and Neurosurgery Manuel Velasco Suarez Mexico city México; ^44^ Pontificia Universidad Javeriana PhD Program of Neuroscience Bogotá Colombia; ^45^ Hospital Universitario San Ignacio Centro de Memoria y Cognición Intellectus Bogotá Colombia; ^46^ Gerosciences Center for Brain Health and Metabolism (GERO) Santiago Chile; ^47^ Memory and Neuropsychiatric Center (CMYN) Neurology Department Hospital del Salvador & Faculty of Medicine University of Chile; ^48^ Neuropsychology and Clinical Neuroscience Laboratory (LANNEC) Physiopathology Department – Institute of Biomedical Science Neuroscience and East Neuroscience Departments Faculty of Medicine University of Chile Santiago Chile; ^49^ Neurology and Psychiatry Department Clínica Alemana‐University Desarrollo Santiago Chile; ^50^ College of Nursing University of Iowa Iowa City Iowa USA; ^51^ Laboratorio de Enfermedades Neurodegenerativas Instituto de Neurociencias‐Fleni‐CONICET Buenos Aires Argentina; ^52^ Cognitive and Behavioral Neurology Unit Hospital das Clinicas University of São Paulo Medical School São Paulo Brazil; ^53^ Department of Neurology University of California San Francisco San Francisco California USA; ^54^ Hospital Isidro Ayora Loja Ecuador; ^55^ Hospital Moinhos de Vento Porto Alegre Brazil; ^56^ Department of Pharmacology Universidade Federal do Rio Grande do Sul Brazil; ^57^ Graduate Program in Biological Sciences: Biochemistry; Pharmacology and Therapeutics Universidade Federal do Rio Grande do Sul Brazil; ^58^ UK Dementia Trials Network Institute of Neurology University College London London UK; ^59^ Alzheimer's Association Chicago Illinois USA

**Keywords:** aging, Alzheimer's disease, brain health, dementia, Latin America

## Abstract

Latin America is undergoing rapid population aging alongside a rising burden of dementia. While the region holds substantial potential for dementia risk reduction, challenges remain, such as delayed diagnoses, limited access to specialized care and biomarker testing, persistent stigma, and deep‐rooted structural inequities. To address these gaps and foster regionally informed solutions, the Alzheimer's Association convened the 2025 Alzheimer's Association International Conference (AAIC) Satellite Symposium in Lima, Peru, on May 14–15, in collaboration with the Global Brain Health Institute (GBHI) and the Atlantic Fellows for Equity in Brain Health. The meeting aimed to bring core elements of the global AAIC meeting to regional Latin American settings, recognizing that national and cultural contexts demand tailored approaches to dementia prevention, risk reduction, treatment and care all aimed at promoting brain health in the region. This manuscript synthesizes the symposium's key discussions, scientific advances, and opportunities for collaboration across the region.

## Highlights

- The 2025 Alzheimer's Association International Conference (AAIC) Satellite Symposium spotlighted regionally driven dementia research across Latin America.
- Dementia research in Latin America is expanding across epidemiology, diagnostics, therapeutics, and care systems.
- Regional collaboration and culturally grounded strategies are essential for equitable dementia prevention and care.

## 1 BACKGROUND

Latin America is experiencing a demographic transition with rapidly aging populations.^1^, ^2^ By mid‐century, Latin America and the Caribbean are projected to be one of the world's fastest‐aging regions in the world, with about 138 million people aged 65 years or older (≈19% of the population).^3^ This demographic shift coincides with a high and increasing prevalence of dementia risk factors, placing the region at the forefront of a growing public health challenge. In 2013, approximately 7.8 million people were living with dementia in the region and these numbers are projected to reach 27 million by 2050.^4^ Despite this, public awareness about dementia is low, diagnosis and treatment are often delayed, and social stigma persists.^5^, ^6^ Limited access to trained health professionals, and to diagnostic and therapeutic resources further hinder adequate care.^5^, ^7^ Consequently, dementia remains underdiagnosed, underfunded, and under‐prioritized across much of the region.

Since 2015, the Alzheimer's Association International Conference (AAIC) Satellite Symposia have convened across the world to spotlight regionally led dementia research, build research capacity, and foster international collaboration in addressing the global burden of Alzheimer's disease (AD) and AD‐related dementias (ADRD). AAIC Satellite Symposia recognizes that the unique contexts of individual countries demand country‐ and culturally‐specific approaches to dementia prevention, risk reduction, treatment and care all aimed at promoting brain health. In 2025, the AAIC Satellite Symposium was held in Lima, Peru, in partnership with the Global Brain Health Institute (GBHI) and the Atlantic Fellows for Equity in Brain Health. The meeting brought together the Latin American community of researchers, clinicians, patients, caregivers, and public health leaders in dementia and brain health to discuss emerging trends, tackle challenges, and drive innovation within the region. This manuscript synthesizes the key discussions and insights that emerged from the meeting, highlighting advances in science, gaps in knowledge, and opportunities for collaboration across Latin America.

## 2 EPIDEMIOLOGICAL INSIGHTS ON DEMENTIA RISK AND PREVALENCE IN LATIN AMERICAN AND LATINO POPULATIONS

The prevalence of dementia in Latin American countries seems to be higher than in most high‐income countries.^8^ Moreover, evidence from high‐income countries suggests a declining trend in dementia incidence, potentially driven by increased educational attainment and improved cardiovascular health.^9^, ^10^, ^11^, ^12^, ^13^, ^14^ However, cross‐national comparisons of dementia incidence trends remain limited in the Global South—including Latin America, Africa, and countries in south, east, and southeast Asia^15^—where context‐specific determinants and risk factors may differ significantly.

In Latin America, findings from the 10/66 Dementia Research Group show contrasting trends in dementia prevalence across Cuba, Dominican Republic, Mexico, Peru, and Puerto Rico. Using standardized data from the first (2003–2006) and third (2016–2019) waves of the 10/66 population‐based community surveys among individuals aged ≥65 years,^16^, ^17^ age‐standardized prevalence rates for 2025 were estimated using country‐specific United Nations projections. Dementia prevalence remained stable in Cuba and Dominican Republic, but marked increases (+5%) were observed in Mexico, Peru and Puerto Rico. Furthermore, despite modest gains in educational attainment between survey waves, the prevalence of modifiable cardiovascular and lifestyle‐related risk factors remained high or increased in several countries. Other studies showed that structural inequalities and social determinants of health outperform classical risk factors in the region^2^, ^18^ and are associated with accelerated brain aging.^19^ These findings suggest that dementia prevalence trends in Latin American countries diverge from those reported in high‐income settings, and highlight the substantial potential for risk reduction of cognitive impairment and dementia in Latin America and the Caribbean.

Population attributable fraction (PAF) is often used to estimate the number of dementias that could be prevented by eliminating modifiable risk factors. However, most PAF estimates have been derived from meta‐analyses of observational studies conducted in high‐income countries.^20^ Early estimates from the 10/66 Dementia Research study in six Latin American countries (Cuba, Dominican Republic, Mexico, Peru, Puerto Rico, and Venezuela) reported a higher proportion of dementia cases attributable to modifiable risk factors.^21^ More recent analysis incorporating nationally representative data from seven Latin American countries (Argentina, Bolivia, Brazil, Chile, Honduras, Mexico, and Peru) confirmed the trend of growing prevalence, with a pooled regional PAF of 54%.^22^ These analyses also revealed substantial heterogeneity in the distribution and impact of individual risk factors. For instance, low educational attainment contributed more significantly to dementia risk in Bolivia and Brazil, while countries like Argentina and Chile had educational profiles closer to global norms. Similarly, the contribution of hypertension varied widely, with a greater burden observed in Argentina and Chile.^22^

Additional within‐country analyses revealed substantial heterogeneity in the distribution of dementia risk factors by sex and income. In Argentina, for example, excessive alcohol consumption and smoking contributed more significantly to dementia risk among men, while social isolation contributed more substantially to dementia risk among women.^23^ Lower‐income groups exhibited greater risk from limited education, hypertension, obesity, and physical inactivity, whereas social isolation was more prominent in higher‐income populations.^23^ Research in Chile showed that subpopulations exposed to adverse social determinants of health exhibit a higher prevalence of cardiometabolic risk factors. Furthermore, individuals with depression and those with elevated cardiometabolic risk demonstrate a greater accumulation of dementia‐related risk factors, highlighting the clustering of vulnerabilities across biological and psychosocial domains.^24^

Together, these findings highlight the heterogeneity of dementia risk profiles not only across but also within countries, emphasizing the need for regionally tailored prevention strategies that account for this heterogeneity.^21^, ^25^ The exposome framework offers a useful lens for this work, as it captures the cumulative impact of genetic, biological, environmental, and social factors over the life course.^25^, ^26^, ^27^, ^28^ In Latin America, where socioeconomic inequality and under‐resourced health systems prevail, the social exposome framework^27^ may provide a more comprehensive lens^28^ than conventional single‐determinant models.^29^

Emerging evidence in Latin America suggests that factors such as educational attainment, quality of education, socioeconomic inequality, and environmental exposures have a stronger association with brain structure and function than in higher‐income countries.^30^, ^31^, ^32^ It has also been reported that individuals in Latin America—especially women with AD—have older brain ages compared to those in non‐Latin American regions, a disparity associated with disproportionate exposure to pollution, healthcare inaccessibility, and structural inequality.^19^, ^24^ Moreover, disparities in mental health, cardiometabolic burden, and education predict cognitive outcomes more strongly than age in Latin America, unlike in wealthier regions.^2^ These findings emphasize that diverse exposures—ranging from early‐life adversity to midlife health conditions and environmental inequality—accumulate over time, shaping long‐term brain health trajectories and increasing dementia vulnerability in structurally disadvantaged populations. Therefore, adopting a social exposome framework could help integrate neurobiological, environmental, and policy‐relevant insights to develop systemic, equity‐focused approaches for dementia prevention. Such approaches should begin early in life^33^ and address structural conditions that drive vulnerability across the life course.

### 2.1 Dementia research on indigenous and hardliest‐reach populations in Latin America

Dementia research among Indigenous peoples is critically underdeveloped, despite the presence of over 42 million Indigenous individuals across the region, primarily concentrated in Mexico, Peru, Bolivia, and Guatemala.^34^ Demographic transitions, marked by increased life expectancy, urbanization, and rising chronic conditions such as hypertension, diabetes, and obesity, are converging with low educational attainment and socioeconomic disparities to elevate dementia risk among predominant Indigenous groups.^35^, ^36^

Although most evidence comes from high‐income countries, a few Latin American studies offer crucial insights.^37^, ^38^ In Mexico, dementia prevalence among the Otomí reached 12.3% using Diagnostic and Statistical Manual of Mental Disorders, 4^th^ Revision (DSM‐IV) criteria.^39^ Conversely, among the Tsimane and Moseten in the Bolivian Amazon, prevalence was remarkably low (0.6%–1.2%), attributed to subsistence lifestyles and low cardiometabolic risk.^40^

Genetic studies further underscore population‐specific differences in dementia risk. While some studies have suggested that apolipoprotein E (APOE) ε4 allele may confer a reduced risk of AD in Hispanic populations compared to non‐Hispanic White populations,^41^ other evidence show that the APOE ε4 allele confers a markedly higher risk for AD among populations with high Amerindian ancestry compared to non‐Hispanic White populations with predominantly European genetic ancestry. In Peruvians, who possess up to ∼80% Amerindian ancestry, the ε4‐associated odds ratios for AD range from 5 to 6, significantly exceeding those typically reported in non‐Hispanic White populations.^42^ These findings, replicated across independent Peruvian cohorts, indicate that local Amerindian genetic background around the APOE locus may amplify ε4‐related risk, whereas global ancestry alone does not explain this difference.^43^

In Brazil, a study in riverine Amazon communities found a dementia prevalence of 12.3% among those aged 65 years and older, linked to age and low education.^44^ Furthermore, in a rural Indigenous group from the Brazilian Amazon, around 43.3% of adults over 50 screened positive for cognitive impairment and dementia, which is a high prevalence potentially attributed to assessment bias.^45^ To address this, researchers in collaboration with community members from a multiethnic urban Indigenous village in Manaus, Amazon, validated the Brazilian Indigenous Cognitive Assessment (BRICA), a culturally grounded tool with high sensitivity (94.4%) and specificity (99.2%).^46^ In that community, 12.8% of individuals aged 50 and older were classified as having dementia, with age and low education as likely contributing factors.^47^ Community engagement proved essential for data collection, trust‐building, and tool development. Barriers such as geographic isolation, historical mistrust, and lack of culturally appropriate assessments were overcome through local partnerships, training, and inclusive funding models.^46^, ^47^

Together, these findings highlight the need for inclusive, community‐led, and culturally grounded approaches to dementia and brain health research, prevention and risk reduction in Indigenous Latin American populations. Socially and culturally tailored tools and meaningful engagement with communities are essential to advancing equity in AD/ADRD science. Future efforts should prioritize culturally sensitive methods, strengthen local research capacity, and center Indigenous voices to both reduce disparities and learn from diverse models of cognitive resilience.

### 2.2 Insights on building an epidemiologic cohort of older Latino adults in the United States focused on brain health: the Rush Latino core

Older Latinos, one of the fastest growing segments of the U.S. population, are at an increased risk of dementia but are under‐represented in clinical research studies. To address this gap, the Rush Latino Core of the Rush Alzheimer's Disease Center (RADC) was established in 2015 as a longitudinal cohort study of older Latino adults who enroll initially without dementia and are followed over time until autopsy.

The overall goal of the Rush Latino Core is to support high quality, cutting edge, research projects on the etiology, pathogenesis, diagnosis, treatment, and prevention of AD/ADRD. Led by a team of researchers with expertise in the Latino culture and deep experience working with older adults, the cohort consists of close to 300 older Latinos, representing 9 countries (63% from Mexico; 22% from Puerto Rico). Compared to older non‐Latino White participants in the RADC, Latino participants tend to be slightly younger (mean age 72 years (SD = 7.7), have fewer years of education (12 years (SD = 5), and are more likely to be women (78%). More than half (58%) prefer to be tested in Spanish and about 63% intend to donate their brain at the time of death.

Harmonized with other cohort studies at RADC, there have been a number of studies conducted to understand ethnic differences in trajectories of cognitive function over time and ethnically‐relevant risk factors for cognitive impairment and decline in the cohort. Recruitment and retention followed the NGAGEDD model (Network, Give 1st, Advocate for research, Give back, Evaluate, Design, and Develop) emphasizing cultural sensitivity and community engagement. Key lessons for recruiting older Latinos include the importance of maintaining a consistent presence in the community, hiring bilingual staff who are familiar with the culture and neighborhoods where participants live, and ways to establish and maintain a mutually beneficial relationship based on giving first and giving back. Similarly, retention strategies have included consistent acknowledgement of special occasions and showing of appreciation, wellness calls and visits in between regularly scheduled evaluations, newsletters with study updates and other information, and keeping all touch points personal and allowing flexibility with study protocols.

Together, these initiatives underscore the importance of culturally informed research infrastructures to ensure the inclusion of diverse populations in AD/ADRD research, and to strengthen the evidence base for understanding and addressing dementia in Latino communities in the United States.

## 3 ALZHEIMER'S AND DEMENTIA DIAGNOSIS: DISEASE CHARACTERIZATION AND DIAGNOSTIC TOOLS IN LATIN AMERICA

Globally, AD diagnosis has experienced significant development during the last decades, with advancement in diagnostic tools such as neuroimaging techniques^48^ and molecular biomarkers.^49^ This has enabled earlier and more accurate diagnosis, which is associated with several benefits for patients and caregivers, including allowing for planning for future care^50^ and the possibility of accessing therapeutic options that are most effective at early stages of specific diseases.

Despite these advancements, delayed dementia diagnosis remains common in Latin America. Contributing factors that delay an individual experiencing symptoms from getting diagnosed include limited awareness and stigma related to dementia, as well as insufficient knowledge of the condition among healthcare professionals.^51^, ^52^ While diagnostic practices vary across Latin American countries due to disparities in healthcare infrastructure and access to specialized services, several shared challenges hinder the diagnostic process. These include delayed referral to specialty clinics,^51^ insufficient time to perform clinical evaluations, lack of validated neuropsychological tests for people with low levels of education or different languages and cultural backgrounds,^4^ and limited access to specialized tests, such as molecular biomarkers and genetic testing.^53^

To address these gaps, multiple initiatives are underway, including training programs for healthcare professionals,^54^ neuropsychological testing validation,^55^ and research collaboration that can help understand the role of diagnostic tools in the Latin American population.^56^ This section highlights advances in neuropathology, imaging, fluid biomarkers, cognitive assessments, and genetic counseling underscoring their critical importance for the effective implementation of AD/ADRD diagnostic strategies across the region.

### 3.1 The role of neuropathology

Late‑life brains rarely host a single neurodegenerative process. Analyzing >2000 autopsies from the Biobank for Aging Studies, an ethnically diverse brain bank in São Paulo, Brazil, it was shown that age‐related tau astrogliopathy, argyrophilic grain disease, limbic‑predominant TDP‑43 encephalopathy, and Lewy body disease routinely co‑occur.^57^ Unpublished neuropathological data from the same cohort revealed further discordance: although 19 % harbored enough pathology to be amyloid‑ and tau‑positive by positron emission tomography (PET)/cerebrospinal fluid (CSF) based biomarkers, had they been tested, isolated cortical tau was far more common than isolated amyloid, which is in contrast with biomarker data. These findings reinforce the limited sensitivity of current amyloid‑ and p‑tau–centric assays to low‑to‑moderate burdens and caution against linear models of AD progression based on biomarkers only. Ancestry‑ and geography‑based heterogeneity in neurodegenerative disease pathology remains understudied; most reference datasets come from high‑income, white populations, constraining diagnostic thresholds and equitable drug development. These insights call for representative autopsy‑biomarker programs to guide equitable disease characterization, diagnosis and care.

### 3.2 Cognitive assessment

Neuropsychological assessment is fundamental to the diagnosis, monitoring of disease progression and stage as well as research of AD/ADRD. Several brief measures show promise for use in Latin American settings,^58^ yet most comprehensive cognitive instruments (i.e., non‐screeners) have been validated primarily in high‐income countries, limiting their applicability in global populations.^59^ While several initiatives have been launched, such as the National Institute on Aging Harmonized Cognitive Assessment Protocol (HCAP), several challenges remain, including poor performance of HCAP executive and spatial tasks in low‐ and middle‐income countries (LMICs).^60^ Similarly, the National Alzheimer's Coordinating Centers Uniform Data Set is confined to English and Spanish and includes language‐dependent measures such as Letter Fluency, limiting use in non‐alphabetic languages.^61^

To address this gap, the GBHI launched the Harmonized Multidomain Cognitive Assessment for Diverse Populations Project in 2023. This initiative aims to develop and culturally adapt a globally harmonized, copyright‐free battery and generate normative data in LMICs.^62^ It builds on previous work identifying major barriers in these settings, including poor cultural adaptation, educational and linguistic biases, low sensitivity to early decline, and a lack of robust norms.^63^ Test selection focuses on minimal linguistic and educational demands, culturally relevant stimuli, and minimal floor and ceiling effects. Adaptation followed gold standard guidelines including qualitative methods and community engagement.^64^ Data collection is underway in Ethiopia, Kenya, Botswana, and Israel using harmonized criteria and shared data management systems. Early results show strong protocol adherence, cultural relevance of the tests, and normal distribution of test scores, signaling promise for global research and clinical use.

It is important to note that cognitive assessment should be complemented by culturally and contextually appropriate measures of functional ability, given its role in the clinical evaluation and progression of dementia. In this context, another regional effort in Latin America has focused on the validation of instruments for assessing functional ability that are culturally and contextually appropriate for diverse populations. This work is particularly significant given the pivotal role of functional ability in the clinical diagnosis of dementia and the substantial influence of sociocultural factors on its evaluation and interpretation.^65^, ^66^

### 3.3 Brain imaging

Individuals from Latin America and the Caribbean remain underrepresented in neuroimaging research, despite its rich genetic, cultural, and environmental diversity. This limits the generalizability of findings and hampers the development of culturally and biologically sensitive brain models.^67^, ^68^

Recent initiatives have begun addressing this gap. Key examples include the Multi‐Partner Consortium to Expand Dementia Research in Latin America (ReDLat),^56^ which has screened over 5000 individuals in 12 countries using harmonized neuroimaging and clinical protocols; the BrainLat initiative,^69^ the EuroLaD‐EEG consortium, Alzheimer's Disease Neuroimaging Initiative (ADNI‐Argentina),^70^ and the Latin American Initiative for Lifestyle Intervention to Prevent Cognitive Decline (LatAm‐FINGERS, multisite).^71^ These efforts have generated multimodal imaging datasets—EEG, magnetic resonance imaging (MRI), functional MRI (fMRI), and PET (less frequently)—in cognitively unimpaired and clinical populations (mild cognitive impairment, AD, frontotemporal lobar degeneration). Multicenter studies from Latin America and the Global North (high‐income, industrialized countries, primarily in North America and Europe), reveal regional differences in brain structure and connectivity, shaped by clinical, demographic, genetic ancestry, and macro‐social factors. Lower connectivity has been linked to structural inequality^32^ and limited education.^30^ Brain age clocks show that factors such as structural inequality, pollution, and health disparities predict accelerated brain aging in the region.^19^ Moreover, these disparities have been linked to fundamental mechanisms associated with pathological brain aging, including excitatory–inhibitory balance, synaptic plasticity, and mesolevel brain efficiency.^72^, ^73^

Emerging generative modeling approaches enhance brain‐aging research in Latin America by capturing complex, region‐specific patterns and improving individualized predictions.^74^ Much more is required to assess the external and intrinsic sources of heterogeneity observed across Latin American populations, and to link whole‐body health and the exposome to computational mechanisms associated with multimodal brain signals.^75^ Barriers such as limited funding, infrastructure, and access to high‐resolution imaging persist. Strengthening local capacity and integrating biological with social data are critical for expanding representation and informing public health strategies.

### 3.4 Blood‐based biomarkers in Brazil

Biomarkers are emerging as essential tools for improving the diagnosis and characterization of AD/ADRD. Their integration into research and clinical practice worldwide is poised to accelerate the shift toward earlier and more precise detection of disease‐related pathophysiology.^76^, ^77^, ^78^, ^79^

In Brazil, biomarker research efforts have included CSF, plasma, MRI, and PET modalities. However, fewer than 500 individuals have been assessed using fluid biomarkers, while over 2000 have participated in imaging‐based studies, predominantly MRI.^15^ Furthermore, although four CSF biomarkers (Aβ40, Aβ42, total tau, and phosphorylated tau [p‐tau]‐181) have been approved for clinical use in Brazil since 2014, and amyloid PET since 2022, these tools are not available within the Brazilian Unified Health System (locally called *Sistema Único de Saúde*, SUS), which serves more than 160 million individuals. Barriers related to infrastructure, cost, and procedural complexity have hindered broad implementation of these biomarker tools.

Recent advances in blood‐based biomarkers (BBMs) could offer a promising alternative for scalable AD/ADRD diagnostics in Latin America and the Caribbean.^80^, ^81^, ^82^ Notably, two independent studies conducted in different Brazilian regions assessed the diagnostic performance of BBMs (p‐tau217, p‐tau181, Aβ40, Aβ42, neurofilament light [NfL], and glial fibrillary acidic protein [GFAP]) against CSF biomarkers.^83^, ^84^ Both studies demonstrated high accuracy of p‐tau217 in distinguishing cognitively unimpaired individuals from those with AD and in predicting Aβ pathology and conversion to dementia, with similar areas under the curves despite differences in participant education levels and regional characteristics.

Together, these findings indicate that the diagnostic accuracy of BBMs in Brazil aligns with results reported in other international studies. In recent years, the Ministry of Health has prioritized the development of cost‐effective biomarker strategies for integration into SUS. With multiple public and private initiatives underway, the generation and harmonization of large‐scale biomarker data will be essential for enabling equitable, scalable strategies for dementia prevention, diagnosis, and care across Brazil and the broader Latin American region. While Brazil has taken a leading role in advancing BBM research, most other countries in the region remain in earlier stages of development.

### 3.5 The role of genetic counseling and testing

Genetic counseling and testing (GCT) represent an emerging yet essential component of dementia diagnosis and management in Latin America. Although genetic testing is particularly important for families affected by Autosomal Dominant Alzheimer's Disease (ADAD), there remains significant debate regarding the use of susceptibility or risk alleles, most notably the APOE ε4 variant, as part of clinical testing. Despite its strong association with Alzheimer's disease in some populations, APOE ε4 is neither necessary nor sufficient for disease development, and numerous studies caution against its diagnostic or predictive use. Concerns include the potential for misinterpretation of individual risk, underestimation of vulnerability leading to false reassurance, overestimation of disease probability, and possible discrimination in health insurance or employment contexts.^85^, ^86^

Latin American countries continue to face major capacity and training limitations in genetics and genetic counseling, posing challenges for the integration of these tools into dementia care. To address the absence of standardized services, researchers established the PRograma de Asesoramiento Genético para América Latina (PRAGA), a regional initiative that developed a harmonized and culturally adapted protocol for ADAD genetic counseling, tailored to the sociocultural and economic realities of Latin American families.^87^ This framework integrates psychological assessment, evaluation of support networks, and consideration of vulnerabilities to ensure safe and equitable delivery of GCT.^88^, ^89^

Data from Argentina and Colombia were used to pilot the PRAGA protocol. The aim was to evaluate the psychosocial impact of genetic testing on asymptomatic individuals within ADAD families.^90^ In a non‐randomized controlled trial with 122 eligible participants, 97 completed the GCT process and 87 chose to learn their genetic status. Measures of anxiety and depression showed no clinically significant differences between those who learned their status and those who did not, nor between mutation carriers and non‐carriers. These results demonstrate that the GCT protocol effectively managed psychological impacts in ADAD families and was positively received, demonstrating the importance of culturally adapted GCT protocols.^91^, ^92^

Importantly, this initiative highlights the feasibility of implementing standardized the GCT protocol in Latin America, demonstrating that it is well tolerated and mitigates psychological distress among participants. Long‐term follow‐up studies are now needed to assess the border public health impacts of GCT and use of GCT for treatment options.

## 4 TREATMENTS AND CLINICAL TRIALS

Latin America remains markedly underrepresented in AD/ADRD clinical trials. A study published in 2013 found that from 715 clinical trials, Latin America was included in only 34 studies (4%). A more recent review found that in a 21‐year study period, 1237 unique AD/ADRD disease‐modifying therapies have been tested in 74 countries (total of 3467 trials). Of these, only 405 (12%) were conducted in upper‐middle income (*n* = 333, 10%), and low‐middle income (*n* = 68, 2%) countries.^93^, ^94^, ^95^ This limited geographic distribution of trials restricts both access to experimental therapies and the relevance of generated evidence to local populations.

A major limitation of existing research practices is considering Latinos a single homogeneous group. In reality, Latin America is a highly diverse region, with substantial heterogeneity in admixture proportions, genetic risk factors, and environmental exposures across populations.^94^ This lack of population‐specific evidence limits the interpretability and generalizability of trial findings for the region.

This section highlights emerging advances in AD/ADRD treatments and clinical trials in Latin America, spanning both pharmacological and non‐pharmacological advances. It covers advances in non‐pharmacological interventions, the implementation challenges of anti‐amyloid therapies as well as the promise of next‐generation therapeutic strategies targeting tau pathology and brain hormonal signaling.

### 4.1 Non‐pharmacological interventions: LatAm‐FINGERS

The Latin American Initiative for Lifestyle Intervention to Prevent Cognitive Impairment (LatAm‐FINGERS) is the first non‐pharmacological multicentre randomized clinical trial designed to prevent cognitive impairment in Latin America. Its main objectives are to investigate the feasibility of implementing the Finnish Geriatric Intervention Study to Prevent Cognitive Impairment and Disability (FINGER) multi‐domain lifestyle intervention in the Latin American context, and to compare the efficacy of a structured multidomain lifestyle intervention against a more flexible lifestyle intervention in improving global cognition.

In this 2‐year randomized clinical trial, an external harmonization process was carried out to follow the FINGER^96^ and the Study to Protect Brain Health Through Lifestyle Intervention to Reduce Risk (US‐POINTER) models,^97^ and internal harmonization was performed to ensure that the study was feasible and comparable across the 12 participating countries. The target sample size is 1200 participants (100 per country). Eligible participants are older adults at risk of dementia—characterized by advanced age, slightly below average cognitive performance, and poor cardiovascular risk profile—identified using the CAIDE scale.

Participants are randomized into two groups: the Systematic Intervention Group (SIG), which receives: (1) aerobic exercise, (2) adapted Mediterranean diet (adapted MIND), (3) social and cognitive stimulation, and (4) Health counseling based on guidelines to control cardio metabolic risk factors; and the Flexible Intervention group (FIG), which received regular health counseling only. Neuropsychological, physical and functional evaluations, as well as laboratory analyses, are conducted at baseline and every 6 months.

Preliminary results show that 1926 adults aged ≥60 years were screened, and 1243 met inclusion criteria and were randomized. Of these, 1167 completed baseline evaluation – 563 in the FIG and 570 in the SIG. Across groups, participants were demographically similar: average age 67, predominantly women, average 13 years of education, mean Mini‐Mental State Examination score of 27, and mean CAIDE score of 8. These participants have now completed the 2‐year intervention and entered the follow‐up phase, with full outcome analyses forthcoming.

### 4.2 Pharmacological interventions

#### 4.2.1 Anti‐amyloid therapies

The underrepresentation of Latin American populations in clinical research has important implications for anti‐amyloid therapies. This lack of representation limits the ability to draw conclusions from subgroup analyses of pivotal phase 3 trials. For example, in phase 3 trials, lecanemab included only 215 Hispanic/Latino participants (vs. 1457 non‐Hispanic participants), while donanemab included just 45 Hispanic/Latino participants (vs. 868 non‐Hispanic participants). These small sample sizes preclude adequately powered analyses and make it difficult to determine whether treatment effects observed in the overall population apply to Hispanic/Latino subgroups.^98^, ^99^

**FIGURE 1:**
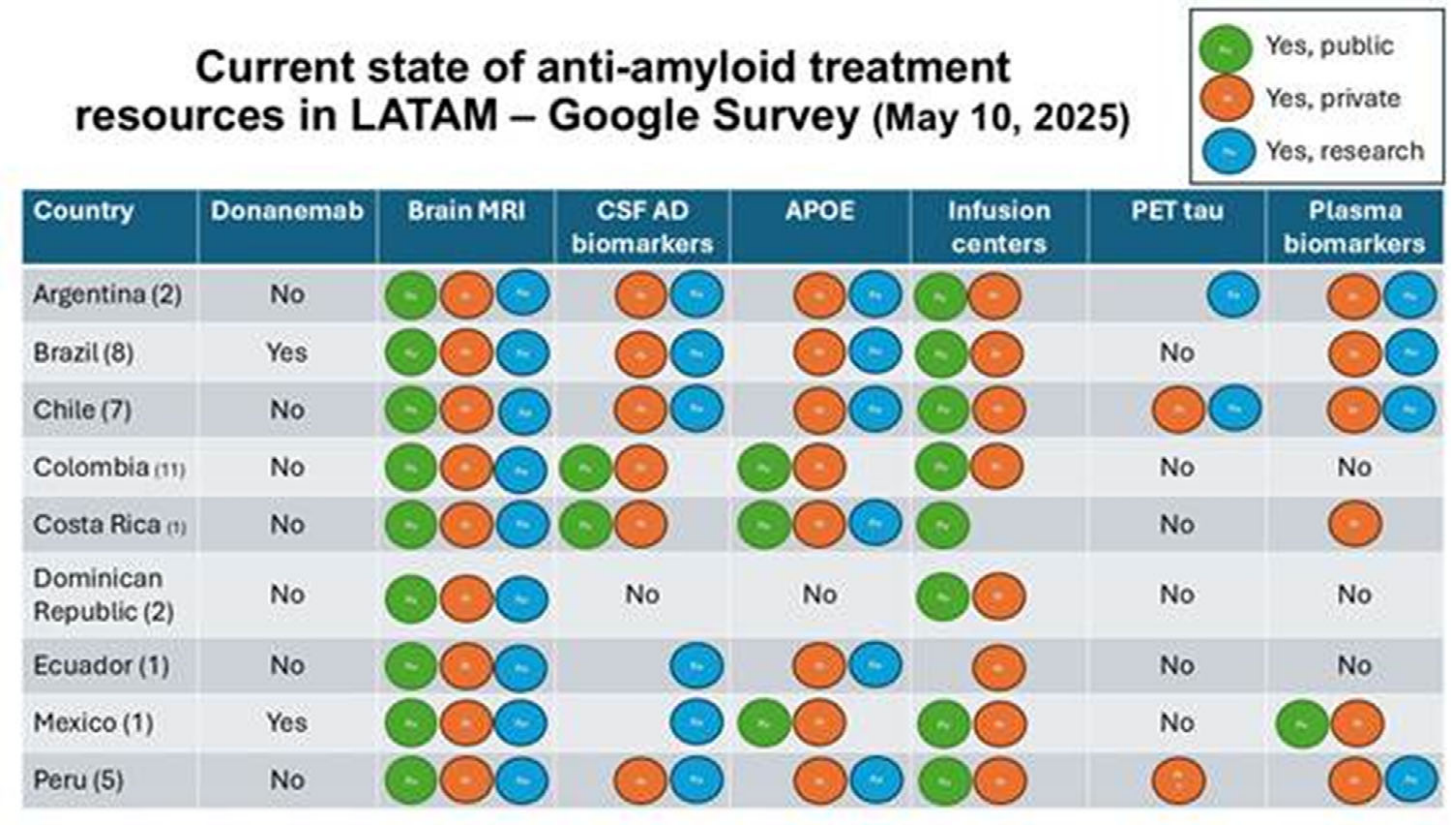
State of anti‐amyloid treatment resources in Latin America as of May 1, 2025.

The safe and effective use of anti‐amyloid therapies in Latin America presents further challenges. According to the recommendations from the Brazilian Academy of Neurology, several resources are essential for the safe use of anti‐amyloid therapies: dementia specialists (neurologists, geriatricians or geriatric psychiatrists) to evaluate treatment indications and contraindications; access to brain MRI before and during treatment; biomarkers tests to confirm amyloid pathology (such as CSF biomarkers or amyloid‐PET); APOE genotyping; specialized infusion centers; and intensive care unit availability for managing adverse events such as amyloid‐related imaging abnormalities (ARIA).^93^ The availability of these resources varies widely across Latin American countries, further contributing to inequities in access (Figure 1).

The costs of anti‐amyloid therapies add another barrier. In the United States, the annual list price of lecanemab is approximately $26,500 and $32,000 for donanemab.^100^ Cost‐effectiveness studies incorporating quality‐adjusted life‐years (QALYs) and direct medical costs suggest that lecanemab could be considered cost‐effective in some settings if priced at less than $5100 annually and donanemab at $20,000 or less.^101^, ^102^ However, even these reduced thresholds are far beyond the means of most Latin American populations. For example, Brazil's minimum monthly wage in May 2025 was approximately $265, meaning even families in the highest income class (≥15 minimum wages/month) would face prohibitive out‐of‐pocket expenses.

Regulatory approval status also varies across the region. Donanemab was approved by the Brazilian Health Regulatory Agency (ANVISA) in April 2025, although it remains unclear whether the country's public health system (SUS) and private insurers will cover the cost. Currently, families may import the medication at their own expense. In Mexico, the Federal Commission for the Protection Against Sanitary Risk (COFEPRIS) authorized lecanemab in December 2024 and donanemab in April 2025 and are now available in the private sector; however, questions remain regarding their reimbursement.

In other countries, such as Argentina, the drugs can be imported under compassionate use programs, with estimated annual costs of around $60,000, entirely borne by patients and families. In Chile, neither drug has been formally approved, but importation is possible, and approximately 20% of the population with private health insurance may face uncertain coverage decisions—although the number of individuals covered by private health insurance (Isapres) in Chile is declining due to the ongoing challenges within the private health sector. In Peru, lecanemab is not expected to be used, and donanemab awaits a feasibility study by the manufacturer. The costs related to drugs will probably have to be covered by patients/families or private insurance companies. These considerations reflect the landscape as discussed during the AAIC Satellite Symposium in Lima, Peru, in May 2025.

Taken together, the implementation of anti‐amyloid therapies in Latin America faces significant obstacles,^93^, ^94^ including limited representation of regional populations in clinical trials, the high cost of medications and related diagnostic procedures, shortages of trained medical professionals and specialized infrastructure resources, and regulatory delays or inefficiencies. Addressing these barriers is critical to ensure equitable access to emerging disease‐modifying therapies and to avoid exacerbating existing disparities in dementia care across the region.

While these challenges illustrate the barriers to implementing anti‐amyloid therapies in Latin America, research and innovation continue to expand globally toward new therapeutic targets. Among these, tau‐targeting approaches and strategies aimed at restoring brain hormonal signaling are among the promising avenues. Although these therapies remain in early clinical development, understanding their mechanisms and progress is essential, as their eventual introduction into clinical practice will raise many of the same issues of access, infrastructure, and cost that are already evident with anti‐amyloid agents.

#### 4.2.2 Novel tau‐targeting therapies – antisense oligonucleotides

Tauopathies are a group of neurodegenerative disorders defined by abnormal tau aggregation and its progressive spread throughout the brain. Across these conditions, the burden and distribution of tau pathology closely correlate with symptom severity and clinical progression, making tau a compelling therapeutic target. *Post mortem* data in individuals treated with aducanumab or AN1792 (an amyloid vaccine) have further underscored this by showing continued tau accumulation in spite of amyloid reduction.^103^, ^104^ These findings emphasize that directly addressing tau could help modify disease courses in tauopathies.

Several strategies are being explored, including immunotherapy, aggregation inhibition and gene silencing.^105^ Among these, gene silencing using antisense oligonucleotides (ASOs) has shown promising results in reducing tau production and accumulation. For example, BIIB080, an ASO targeting the microtubule‐associated protein tau (MAPT) gene demonstrated reduced tau accumulation, improved survival rate and behavior, and reversal of tau deposition in ASO‐treated mice.^106^ Subsequently a phase I multiple ascending dose placebo‐controlled trial in mild AD^107^ demonstrated a dose dependent, sustained reduction in CSF levels of total and p‐tau to around 40% of levels at baseline. Consistent with the animal data, available tau PET data at baseline and end of open‐label extension (OLE) study, showed a clear reduction in tau levels across the whole brain, especially the temporal regions.^108^ This was the first demonstration of robust brain tau lowering, although it was in small numbers, and needs confirming along with assessment of efficacy. To that end, a large phase II trial is underway, with individuals being dosed once every 3–6 months.^108^, ^109^

Furthermore, a first of its kind trial is ongoing to demonstrate the effects of a MAPT ASO on levels of tau in the CSF, and other studies are investigating active transport mechanisms of delivery to, enable the drug to be given intravenously.^110^

Taken together, targeting tau in AD using novel delivery methods alongside new methods such as gene silencing will broaden the portfolio of drugs and optimize chances of success at different stages of AD. Multiple gene silencing therapies are in development and have the potential to enhance therapeutic potential for AD and other dementias. These advances underscore the potential for future therapies for AD beyond amyloid, but they also raise important considerations for the Latin American context. As with anti‐amyloid therapies, ASO‐based interventions will likely require specialized infrastructure, including intrathecal delivery capacity, advanced biomarker testing, and longitudinal imaging, which are currently limited in many Latin American settings.

#### 4.2.3 Brain hormonal signaling as a therapeutic target in AD

Mounting evidence supports the notion that the pathogenesis of AD, and its impact on memory and cognitive function, results from a complex interplay between biological, socioenvironmental and lifestyle factors.^26^ Within this framework, dysregulated brain hormonal signaling has emerged as a particularly important mechanism that may be leveraged for the development of novel therapeutic approaches.

One area of growing interest is the role of insulin and insulin‐like growth factor 1 (IGF‐1) signaling in AD. Since the original report that neuronal insulin signaling is impaired by soluble Aβ oligomers,^111^ several reports have demonstrated that brain signaling by insulin and IGF‐1 is impaired in AD and AD models,^112^, ^113^, ^114^ and that activation of the insulin/IGF‐1 signaling pathway by GLP‐1 agonists prevents neuronal damage and protects memory in AD models.^112^, ^115^, ^116^

More recent studies have focused on additional brain hormonal signaling mechanisms in the context of AD. For example, brain oxytocin was found to be reduced in both in vitro and in vivo models of AD, and its intranasal administration reversed previously established social, object recognition and spatial memory impairments in aged APP/PS1 mice.^117^ Furthermore, the neuroprotective actions of irisin—an exercise‐induced myokine found to be reduced in the brains and CSF of individuals with AD^118^, ^119^, ^120^ – are being investigated to identify the brain receptors and mechanisms through which irisin exerts its beneficial effects against memory decline in AD models.

Taken together, findings over the past 15 years have advanced our understanding of how defective brain hormonal signaling could contribute to AD pathophysiology, supporting its role as a potential mechanism that influences brain resilience and cognitive outcomes. Integrating research on brain hormonal signaling into Latin American translational research agendas could help identify scalable, cost‐effective interventions that complement existing dementia prevention and care strategies.

## 5 DEMENTIA CAPACITY BUILDING IN LATIN AMERICA: PROGRESS AND CHALLENGES

Building capacity to address dementia in Latin America requires coordinated action across several fronts: training and education for health professionals and caregivers; the development of national dementia plans and legislation; reinforcement of healthcare infrastructure for diagnosis, treatment, and long‐term care; and advancing research in epidemiology, biomarkers, genetics, and therapies. However, these efforts are constrained by funding shortfalls, shortages of specialists, limited research infrastructure, cultural barriers, and socioeconomic inequities.^5^, ^6^

Despite these challenges, important and significant progress has been made. Countries such as Argentina, Chile, and Costa Rica have adopted national dementia plans, training programs for primary care providers have expanded, non‐governmental organizations are increasingly active, and specialized centers are emerging.^4^ Moreover, there has been an increase in research collaborative studies and development of local networks. Initiatives such as LatAm‐FINGERS,^71^ ReDLat,^56^ and IMPACT Salud^121^, ^122^ highlight the potential of collaborative research networks in Latin America to drive scientific innovation while strengthening local and regional research capacity.

Looking ahead, sustained progress in dementia care will depend on the implementation of robust national strategies, improved data collection to guide policy, investment in workforce training and healthcare infrastructure, expanded use of telehealth, and strengthened caregiver support. Public–private partnerships and regionally coordinated research led by local investigators will also be crucial.

### 5.1 Health system opportunities

The coronavirus disease 2019 (COVID‐19) pandemic exposed deep‐rooted fragilities across Latin American health systems. Peru, alongside other countries in the region, recorded some of the world's highest excess mortality rates,^123^ highlighting systemic fragilities. Yet disruption also creates opportunities for transformation.^124^ Implementation science offers one pathway forward by examining what works, for whom, and under what conditions, and by helping to translate evidence into real‐world practice. Such translation, however, depends on institutional readiness, operational capacity, and workforce resilience.^125^, ^126^ As demonstrated by the Jamaican early stimulation program, even well‐evidenced interventions can lose impact if not adapted and scaled appropriately.^127^

Three realities underscore the need for systemic change. First, many health systems continue to rely on outdated service models and governance arrangements that restrict their adaptability. Second, reforms are often layered onto legacy structures without redesigning the underlying architecture, creating fragmented services, operational inefficiencies, and provider burnout as staff navigate overlapping mandates. Third, services are frequently developed without input from patients, families, or frontline providers, even as other sectors increasingly prioritize user experience and trust. Under the WHO ICOPE framework,^128^ overcoming these limitations requires aligning services around intrinsic capacity and functional ability, strengthening community‐level integration, and creating multilevel governance structures that support coordinated, person‐centered models of care.

Dementia care exemplifies these systemic shortcomings in Latin America. Managing dementia requires continuity, coordination, and caregiver integration—capacities still rare in many systems.^129^ It reveals whether care is genuinely person‐centered and sustained across time and settings. While care models often include multidisciplinary teams and chronic care orientation, high‐performing high‐quality health systems must also deliver outcomes that matter to people: reliable access to care, respectful treatment, consistent follow‐up, and a sense of trust in the system.^11^, ^130^ Looking ahead, future models should also integrate strategies for risk reduction and brain health promotion, recognizing that effective care encompasses not only management of symptoms but also proactive approaches to delay or prevent disease onset. This aligns with ICOPE's emphasis on promoting intrinsic capacity through primary and community care interventions that support self‐care, maintain functionality, and reduce avoidable dependency.

Ultimately, health system reform in Latin America must extend beyond incremental adjustments, embracing approaches that redesign care around people, strengthen sustainability, and build resilience to adapt to future challenges.

#### 5.1.1 Regional perspectives on dementia national plans and health system responses

National dementia plans in the region show stark regional heterogeneity. Some countries, such as Chile and Costa Rica, have established plans and structures, while others—including Brazil and Mexico—are piloting or finalizing strategies. Argentina, Panama and Peru do not yet have national plans but show strong subnational or civil society leadership. In more fragile systems, efforts are only beginning. Progress toward the WHO's target of 75% global dementia plan coverage by 2025 is hindered by fragmentation between primary, specialty, and long‐term care; weak integration across health and social protection systems; inequities by geography and ethnicity; workforce shortages; and limited registries or reporting.

Nevertheless, successful examples exist. Chile has integrated cognitive impairment pathways into primary care^131^ including an effort to increase capacity building in primary care through a collaboration with the GBHI, the university of Chile and the ministry of health.^132^ Brazil passed a dementia law following strong advocacy coalitions. Mexico's national federation (FEDMA) has advanced policy co‐production across federal, indigenous, and civil society levels. Costa Rica has legislatively backed and budgeted its dementia plan, an exception in the region. These experiences illustrate how civil society can catalyze system reform when governments create enabling conditions.

#### 5.1.2 Methodological approaches to assessing health system responses: the Peruvian example

A health system assessment enables decision‐makers and researchers to better understand and disentangle the complexity of health system structures and processes.^133^ Quantitative approaches provide information on performance, while qualitative approaches capture the perspectives of key actors and the dynamics of their interactions. For this reason, mixed‐method designs are essential for evaluating health system responses. The IMPACT Salud project^134^ applied two qualitative methodologies to assess how health systems respond to people with dementia and their caregivers^122^: the Rapid Assessment Protocol for Insulin Access (RAPIA)^135^ and the Patient Journey approach.^136^

RAPIA, previously used in studies of non‐communicable diseases,^137^ was adapted to interview participants across 11 themes related to the health system at macro (decision‐makers, opinion leaders, organizations), meso (regional authorities, hospital staff), and micro levels (people with dementia with preserved functionality, caregivers, and primary care professionals).^122^ The Patient Journey approach, by contrast, focused on mapping the experiences of people with dementia, their caregivers, and healthcare providers across points of contact within the health system, exploring emotions, time intervals between consultations, and barriers and facilitators to diagnosis and treatment.^122^ Data analysis will be guided by the High‐Quality Health System framework.^130^

Recruitment of people with dementia presented challenges, as individuals identified at primary care facilities often had advanced cognitive impairment, reflecting the common pattern of late diagnosis in these settings. Next steps for IMPACT Salud include completing the analysis and sharing findings with decision‐makers and stakeholders. In addition, three other Latin American countries (Argentina, Brazil, and Colombia) will conduct similar health system readiness studies to enable cross‐country comparisons and contextual analyses.

### 5.2 The role of clinician training and primary care

Knowledge about dementia among healthcare professionals in Latin America remains uneven. A population‐based study involving 1414 individuals, better knowledge was observed among those with a higher level of education, those under 65 years of age, and among health professionals. Yet misconceptions persisted: more than 70% failed to identify cholesterol and systemic arterial hypertension as risk factors for AD; although 92% agreed with the statement that AD has no cure, and that AD is a type of dementia.^138^ Similar gaps are evident in clinical practice. In Chile, 81% of professionals reported feeling unprepared to treat people with dementia,^52^ while in Peru, only 39% of physicians considered themselves capable of diagnosing and managing people with dementia.^139^

These shortcomings contribute to high rates of underdiagnosis. A systematic review and meta‐analysis of 23 studies from 1988 to 2015 reported underdiagnosis ranging from 54% to 93%, while Brazil's National Report on Dementia (RENADE) estimated the rate at about 88%.^140^ Contributing factors include insufficient training, persistent stigma, and cultural beliefs that conflate cognitive decline with normal aging.

Improving clinical capacity requires dementia education to be embedded throughout medical training, from undergraduate curricula to residency and specialist programs, supported by academic society guidelines and a multidisciplinary framework. Theoretical teaching must be complemented by practical, scenario‐based learning and direct patient exposure, emphasizing competency‐based, person‐centered care. While online and face‐to‐face formats can both be effective, empathy and clinical judgment are best cultivated through hands‐on experience.^141^, ^142^, ^143^

Importantly, primary care physicians (PCPs) are pivotal in translating these competencies into practice. Guided by the WHO/PAHO Integrated Care for Older People (ICOPE) framework, PCPs serve as the cornerstone of dementia care, promoting early detection, holistic assessment, and personalized care planning. By supporting shared decision‐making and collaborating with multidisciplinary teams, PCPs help people with dementia maintain autonomy while addressing physical, cognitive, and psychosocial needs.

Beyond clinical care, PCPs also coordinate health system responses, linking geriatrics, neurology, social care, and community resources to mitigate fragmentation and ensure equitable, context‐sensitive care transitions—an essential feature for progressive conditions like dementia.^144^, ^145^, ^146^ They further advocate for sustainable care models by promoting health education, community‐based rehabilitation, stigma reduction, non‐pharmacological interventions, and palliative care integration, thereby strengthening both quality of care and caregiver resilience.^126^, ^147^, ^148^

Furthermore, PCPs could contribute to generating context‐specific evidence by engaging in research and implementation science. Their involvement helps ensure that interventions are culturally relevant, scalable, and aligned with local systems.^149^, ^150^, ^151^ By combining clinical expertise, leadership, education, and research, PCPs advance equitable dementia care and contribute to resilient health systems that prioritize dignity and person‐centeredness across the dementia journey.

Taken together, comprehensive education equips clinicians with the knowledge, skills, and empathy needed to recognize and manage dementia, while robust primary care systems ensure these competencies are applied consistently in practice. Training should target not only physicians but also non‐medical professionals and be adapted to the specific needs and capacities of primary care teams.^132^ Aligning training with primary care reform represents a cornerstone of equitable and sustainable dementia capacity building in the region.

### 5.3 Training and support of caregivers

Caregivers of people living with dementia are typically classified into two groups: informal caregivers—also known as care partners or unpaid caregivers—are relatives, friends, or others within the person's immediate social network who provide care without formal training or financial compensation. In contrast, are trained professionals who are paid to provide care as part of their occupation. In Latin America and the Caribbean, caregiving is mostly informal, and the role of caregiver is generally assumed by women.^152^ Without support from social and health services, caring for those living with dementia relies largely on household isolated efforts. Caring responsibilities in the region have significant labor and financial constraints impacting the wellbeing and mental and physical health of the caregivers.^152^

Different types of interventions may impact the informal caregivers’ outcomes: (1) interventions for the person living with dementia, such as BPSD management, cognitive stimulation, and physical activity; (2) interventions for caregivers, such as respite, coping skills, psychoeducation, and cognitive‐behavioral therapy techniques; (3) interventions for improving the physical environment, such as improving living space or lights; and (4) dyadic intervention that involves both the caregiver and the person living with dementia, such as case management or multicomponent programs including several of the elements described above.^153^

There is little evidence on the effects of psychosocial interventions on the outcomes of informal caregivers in Latin America and the Caribbean. A recent scoping review found 45 studies conducted in 8 out of the 31 countries in the regions (26%), of which, 38% were randomized controlled trials, and most were conducted in Brazil. In addition, 52% of the interventions evaluated in randomized trials were adapted from programs in other countries, and 65% were multicomponent. Overall, these interventions demonstrated positive effects on caregivers’ depression, quality of life, and burden.^154^

Currently, two interventions have shown positive effects in Latin America and the Caribbean. The first one is the WHO iSupport for Dementia, which has been adapted and tested through a randomized controlled trial in Brazil. The results showed that iSupport significantly reduced burden and anxiety symptoms among informal caregivers. Although depressive symptom scores decreased, the differences were not statistically significant.^155^ The second intervention is the “Providing Care while Caring for Yourself” psychoeducational program, which was adapted from Spain to the Chilean context and evaluated in a randomized controlled trial.^156^ The intervention, based on cognitive‐behavioral therapy and psychoeducational content, effectively reduced dysfunctional thoughts and increased the frequency and satisfaction with leisure activities in Chilean family caregivers of people living with dementia. Decreased burden and depressive and anxious symptoms were observed in caregivers who attended the program; however, these reductions were not statistically significant.

Since Latin America and the Caribbean are highly heterogeneous, they must be systematically documented to ensure replicability and relevance across countries. This applies equally to recruitment and engagement strategies, which should emphasize feasibility and cultural meaning for caregivers and care recipients alike. Ongoing evaluation of such interventions will help clarify what works, for whom, and under which conditions—ultimately improving support for caregivers and people living with dementia across Latin America and the Caribbean.

In summary, multicomponent interventions tend to be the most effective for a range of caregivers outcomes, yet a single approach cannot meet all caregivers’ needs. It is important to assess caregivers’ needs before referring them to an intervention and to continuously assess them throughout the disease trajectory. Future research should adopt more rigorous methodologies (e.g., randomized or pragmatic trials) and implement science frameworks that involve key stakeholders, such as caregivers, people living with dementia, health staff, and policy‐makers.

### 5.4 Research and technology innovations

Research and technology innovations are important components of capacity building, offering scalable tools to strengthen diagnostics, support health promotion, and expand research networks. Emerging approaches in Latin America include digital speech markers and mobile health (mHealth) that hold the potential to advance diagnostic and research capacity across the region.

#### 5.4.1 Digital speech markers

Digital speech markers (metrics of acoustic and linguistic anomalies in verbal production) are gaining momentum in cognitive and dementia research.^157^ Accruing evidence underscores their utility for disease identification, phenotyping, monitoring, and preclinical detection.^158^, ^159^, ^160^, ^161^, ^162^ Thanks to their robustness, automaticity, and cost‐efficiency, these tools are being increasingly incorporated in clinical trials^163^ and in cross‐national efforts, like ADNI and ReDLat. Yet, translation of basic findings into clinical practice remains incipient, calling for implementation breakthroughs at the crossing of science, medical practice, and industry, especially in LMICs, where scalable diagnostic innovations are most urgently needed.

Promisingly, strategic initiatives have been launched. Consider the Toolkit to Examine Lifelike Language (TELL) app, an AI‐driven web‐based speech testing platform. Released by Latin American innovators in 2023,^164^ and revamped in 2025,^165^ TELL offers data collection, encryption, processing, download, and visualization functions, all available for onsite, offline, and videoconference protocols. Via strategic speech tasks, it allows tapping on semantic memory, episodic memory, emotional processing, and motor function.^165^ TELL's reports, plotting participants’ outcomes against normative data, are already informing clinical decision‐making and being incorporated into clinical histories in several institutions worldwide.

TELL's trajectory carries lessons for LMIC‐based translational projects. Key challenges include ensuring sustainable funding, meeting regulatory requisites (which vary widely across countries), and achieving continued presence in relevant events (set mainly in the United States and Europe). These hurdles can be tackled by creating a diverse ecosystem, involving local and international funds, accelerators, clients, and partners committed to digital health. TELL also illustrates actions to boost impact, such as assembling an interdisciplinary team, prioritizing clinical usability, and adapting to regional needs (e.g., by including offline versions for regions with poor Internet connectivity). Further work along these lines can maximize the presence of LMIC innovations in the local dementia landscape.

#### 5.4.2 Technology use for health promotion in Latin America

Chronic diseases, particularly cardiovascular diseases, are the leading cause of disability‐adjusted life‐years (DALYs) in Latin American countries.^166^ Up to 80% of cardiovascular disease risk factors^167^ and 54% of dementia‐related risk factors are potentially modifiable.^22^ However, public health strategies focused on prevention have demonstrated limited effectiveness over recent decades. Key barriers include limited outreach, low population adherence, and significant inequities in access to healthcare.^166^

The WHO has promoted mHealth—the use of portable technologies such as smartphones and smartwatches—as a tool to improve prevention efforts, especially in areas with limited healthcare infrastructure. These devices are widely available and, by enabling access in any location with internet connectivity, they reduce reliance on specialized healthcare services and contribute to resource optimization.^168^ Additionally, their relatively low cost supports scalable implementation.

Nevertheless, mHealth can unintentionally deepen health inequalities, especially among those with limited access to technology or low digital literacy.^169^ Older adults face multiple barriers, including limited digital skills, low broadband access (e.g., only 50% in Chile), physical and cognitive impairments (e.g., presbyopia, slower processing, and reduced motor precision), and a lack of trust in digital tools due to fear of scams or device misuse.^169^

To address these challenges, digital training and user‐centered design are essential. Software should be accessible—featuring large buttons, high contrast, and simple navigation—and culturally adapted. Co‐creation with older adults throughout the development process is crucial to building trust and relevance.^170^

To leverage these opportunities while mitigating risks, researchers in Latin America have developed Neomayor, a mobile application promoting healthy lifestyles and brain health in older adults.^170^ Based on the LatAm‐FINGERS guidelines^71^ and adapted to the Chilean context, Neomayor demonstrated high feasibility and potential cardiovascular benefits in a pilot study.^170^

In summary, mHealth could reduce inequities and increase precision in health promotion in Latin America. Realizing this potential, however, requires initiatives that are culturally tailored, co‐created with older adults, and adapted to the diverse social and technological contexts of the region.^71^, ^171^

## 6 SUMMARY

Cognitive and dementia research across Latin America is experiencing rapid growth, marked by important advances in epidemiology, diagnostics, therapeutics, capacity building, and technology innovation. Epidemiologic findings highlight heterogeneous dementia prevalence trends, the considerable potential for risk reduction through modifiable factors, and the relevance of the exposome framework, alongside insights emerging on under‐studied populations and cohort‐building initiatives among Latino communities in the United States. Advances in diagnosis spans neuropathological comorbidities, multicenter neuroimaging collaborations (e.g., ReDLat), emerging BBMs, culturally adapted cognitive assessments, and regionally tailored genetic counseling and testing. Genetic studies in Latin American populations can provide valuable insights into the genetic architecture of dementias and support the development of culturally and regionally adapted models for genetic counseling and testing. Therapeutics advances include findings from LatAm‐FINGERS and other non‐pharmacological interventions, as well as considerations of barriers to implementing anti‐amyloid therapies, and exploration of early drug development pipelines targeting tau and brain hormonal signaling with potential future applicability in the region. Capacity‐building priorities should center on health system opportunities, clinician education, primary care–anchored models, and implementation science to strengthen person‐centered systems and models for supporting caregivers. IMPACT Salud focuses on dementia as a tracer for high‐quality health systems providing unique potential insights to redefine dementia‐sensitive health systems in the region. Importantly, examples such as Chile suggest that it is feasible to improve the capacity of the health system for dementia care through dementia policies.^4^ Furthermore, digital speech markers and mHealth tools could provide scalable, equity‐focused approaches when co‐created with older adults.

Collectively, these developments illustrate regional momentum, expanding collaborations, and the global relevance of Latin American contributions to equitable dementia and brain health prevention, diagnosis, treatment, and care. Furthermore, they highlight the need for strategies that operate across multiple levels: while some ADRD determinants—such as demographic transitions, health‐system constraints, and structural inequities—may best be addressed through coordinated region‐wide approaches, others such as access to diagnostics and treatments, regulatory environments, and public health priorities may require national or subnational adaptation. Together, these considerations highlight the importance of flexible, context‐sensitive frameworks to effectively translate regional progress into meaningful impact.

## CONFLICT OF INTEREST STATEMENT

All disclosures are based on ICMJE forms for the past 36 months.

Claudia K. Suemoto received funding from the Alzheimer's Association (24CBIDR‐1185483) and FAPESP (2024/03917‐7); payment or honoraria from Lily and Novo Nordisk; and support for attending meetings and/or travel from the Alzheimer's Association. She also participated on a Data Safety Monitoring Board or Advisory Board of Lily, Bazilian Society of Geriatrics and Gerontology and ISTAART.

Diego Aguilar received support from Alzheimer's Disease International.

José A. Avila‐Funes received funding (Award ID: SG‐20‐725707‐ReDLat and RAS Award ID: A135350) and support for attending meetings and/or travel from the Alzheimer's Association.

Sandra Baez received grant funding from the Global Brain Health Institute, Alzheimer's Association, Alzheimer's Society UK, Pilot Awards for Global Brain Health Leaders (Grant Number: GBHI ALZ UK‐ 25‐1289623), and Wellcome Trust (Grant Number: 227012/Z/23/Z); and support for attending meetings and/or travel from the Alzheimer's Association and Universidad de la Amazonía.

Lisa L. Barnes received funding from the NIH (P30AG72975) and is the deputy editor of Alzheimer's & Dementia journal.

Sonia M.D. Brucki received payment or honoraria for lectures and presentations from Roche, Eli Lilly, Novo Nordisk, and Biogen; and served as a member of Advisory Board for Roche, Eli Lilly, Novo Nordisk, Biogen, and Hypera Mantecorp.

Mario Cornejo‐Olivas received subcontract through San Marcos Foundation from NIH‐NIA and GP2/ASAP/MJJF; funding from CHDI; payment or honoraria from International Parkinson and Movement Disorders Society MDS; and support for attending meetings and/or travel from NIH. He also served on the SPC board committee for AAIC Satellite Symposium Lima 2025 and the Executive Committee MDS‐PAS.

Carolina Delgado Derio received funding from the Alzheimer's Association and Agencia nacional de investigación y desarrollo.

Sergio T. Ferreira received funding from the National Institute for Translational Neuroscience (INNT‐Brazil), Conselho Nacional de Desenvolvimento Científico e Tecnológico (CNPq‐Brazil) and Fundação Carlos Chagas Filho de Amparo à Pesquisa do Estado do Rio de Janeiro; and lecture honoraria from Biogen Brazil.

Adolfo M. García is an Atlantic Fellow at the Global Brain Health Institute (GBHI) and is partially supported by the National Institute On Aging of the National Institutes of Health (R01AG075775, 2P01AG019724); ANID (FONDECYT Regular 1250317, 1250091); DICYT‐USACH (032351G_DAS); Agencia Nacional de Promoción Científica y Tecnológica (01‐PICTE‐2022‐05‐00103); and the Multi‐partner Consortium to Expand Dementia Research in Latin America (ReDLat), which is supported by the Fogarty International Center and the National Institutes of Health, the National Institute on Aging (R01AG057234, R01AG075775, R01AG21051, and CARDS‐NIH), Alzheimer's Association (SG‐20‐725707), Rainwater Charitable Foundation's Tau Consortium, the Bluefield Project to Cure Frontotemporal Dementia, and the Global Brain Health Institute. He also holds stocks at TELL Inc.

Lea T. Grinberg received funding from NIH and the Rainwater Charitable Foundation; consulting fees from Guidepoint Inc; payment or honoraria from Medscape Inc and Otsuka Pharmaceutical Development & Commercialization, Inc; support for attending meetings and/or travel from the Alzheimer's Association and the Rainwater Charitable Foundation; and had leadership or fiduciary role in Global Brain Health institute.

Agustin M. Ibanez is funded by ANID/FONDECYT Regular (1250091, 1210176, 1220995); ANID/FONDAP 15150012; ANID/PIA/ANILLOS ACT210096; ANID/FONDAP 15150012; Takeda CW2680521 and the Multi‐Partner Consortium To Expand Dementia Research In Latin America (ReDLat, supported by Fogarty International Center [FIC]) and National Institutes of Health, National Institutes of Aging (R01 AG057234, R01 AG075775, R01 AG21051, R01 AG083799, CARDS‐NIH), Alzheimer's Association (SG‐20‐725707), Rainwater Charitable foundation – Tau Consortium, the Bluefield Project to Cure Frontotemporal Dementia, and Global Brain Health Institute)].

Rosario Isasi received grant funding from the NIH (R01 AG070864 and U19 AG074865) and support for attending meetings and/or travel from the Alzheimer's Association.

Sylvia E Josephy‐Hernandez received payment for lectures from Abbott Pharmaceuticals and Adium Pharmaceutical; and support for attending meetings and/or travel from the Alzheimer's Association.

Jorge J. Llibre Guerra received the following grants: K01AG073526, AARFD‐21‐851415, SG‐20‐690363 and MJFF‐020770.

Mychael V. Lourenco received grants from the Alzheimer's Association, Serrapilheira Institute, Pioneer Science Initiative, Brazilian Ministry of Health, National Research Council – Brazil—CNPq, and Rio de Janeiro State Science Agency – Brazil—FAPERJ; and honoraria for lectures from Novo Nordisk and Biogen. He is a senior editor for reviews for Journal of Neurochemistry.

B. Marcela Mar Meza received a pilot award and support for attending meetings and/or travel from the Alzheimer's Association.

J. Jaime Miranda received the following grant fundings: Alliance for Health Policy and Systems Research (2009/32034, 2012/253750), Bloomberg Philanthropies (grant 46129, via University of North Carolina at Chapel Hill School of Public Health), FONDECYT via CIENCIACTIVA/CONCYTEC, British Council, British Embassy and the Newton‐Paulet Fund (223‐2018, 224‐2018), DFID/MRC/Wellcome Global Health Trials (MR/M007405/1), Fogarty International Center (R21TW009982, D71TW010877, R21TW011740, K01TW011478), Grand Challenges Canada (GMH‐ POC‐0335‐04), International Development Research Center Canada (IDRC 106887, 108167), Inter‐American Institute for Global Change Research (IAI CRN3036), National Cancer Institute (NCI 1P20CA217231), National Council for Scientific and Technological Development (CNPq Brasil 408523/2023‐9), National Health and Medical Research Council (NHMRC 2022566), National Heart, Lung and Blood Institute (NHLBI HHSN268200900033C, 5U01HL114180, 1UM1HL134590), National Institute for Health and Care Research (NIHR 150261, NIHR 150287), National Institute of Diabetes and Digestive and Kidney Diseases (NIDDK K23DK135798), National Institute of Mental Health (NIMH 1U19MH098780), NSW Health, Cardiovascular Elite Postdoctoral Researcher Grants (H23/37663), Swiss National Science Foundation (40P740‐160366), UKRI BBSRC (BB/T009004/1), UKRI EPSRC (EP/V043102/1), UKRI MRC (MR/P008984/1, MR/P024408/1, MR/P02386X/1, MR/X004163/1, MR/X020851/1), Wellcome (074833/Z/04/Z, 093541/Z/10/Z, 103994/Z/14/Z, 107435/Z/15/Z, 205177/Z/16/Z, 214185/Z/18/Z, 218743/Z/19/Z), World Diabetes Foundation (WDF15‐1224) and the World Health Organization (2021/1189041, 2022/1249357). JJM received consulting fees from Pan American Health Organization (2023), Bloomberg Philanthropies (2022‐2023) and Health Action International (2020‐2021). JJM served on DSMB of Nigeria Sodium Study (NaSS); Trial Steering Committee of INTEnsive care bundle with blood pressure Reduction in Acute Cerebral haemorrhage Trial (INTERACT 3); International Advisory Board of Latin American Brain Health institute (BrainLat), Universidad Adolfo Ibáñez (Chile); Consultative Board of Programa de Gastronomía, Facultad de Estudios Interdisciplinarios, Pontificia Universidad Católica del Perú; and Advisory Board of InterAmerican Heart Foundation (IAHF). J.J.M. is co‐Chair of Independent Group of Scientists (IGS), 2023 Global Sustainable Development Report, United Nations; Member of Scientific Expert Committee, Global Data Collaborative for CV Population Health, World Health Federation, Microsoft, and Novartis Foundation; Member of Scientific and Technical Advisory Committee (STAC), Alliance for Health Policy and Systems Research, World Health Organization; Member, of WHO Technical Advisory Group on NCD‐related Research and Innovation (TAG/RI), Noncommunicable Diseases Department, World Health Organization; and Member of Advisory Scientific Committee, Instituto de Investigación Nutricional (Peru).

Claudia Miranda‐Castillo received support from ANID Millennium Science Initiative Program (Chile).

Catherine J. Mummery received a grant from Biogen for investigator led trial of ultrafast MRI in dementia; consulting fees from Biogen, Roche, Lilly, Eisai, Novartis, Neurimmune, MSD, and GSK; honoraria for lectures from Eisai and Lilly; travel and accommodation support for attending meetings from Biogen, Eisai, Roche, and Alector. She served as Lilly Expert advisor in development of clinical programme of siRNA J4T‐MCL‐0LAA; Lilly Member of advisory board on donanemab Trailblazer Fees paid for time; Novartis Member of advisory board on AD drug programme steering committee Fees paid for time; Roche/Genentech Member of advisory board for trontinemab Fees paid for time on advisory board; Eisai—Member of advisory board on UK AUR for Leqembi Fees paid for time on advisory board; and Chair data safety monitoring board Immunobrain. She is Director of NIHR UK Dementia Trials Network and co‐chair of NIHR dementia Translational Research Collaboration

Jose Francisco Parodi Garcia is President of Academia Latinoamericana de Medicina del Adulto Mayor on a pro‐bono basis.

Natalia Pozo Castro received grant funding from Alzheimer`s Association – Global Brain Health Institute – Alzheimer Society (UK).

Hernando Santamaría‐García received grants from NIH, Global Brain Health Institute and Alzheimer's Association.

Rosa María Salinas Contreras received grant funding from the Alzheimer's Association.

Elena Tsoy received grant funding from NIH and the Global Brain Health Institute; payment from American Psychological Association and UC Irvine for lectures, presentations, speakers bureaus, manuscript writing or educational events; and travel support from the Aga Khan University, Washington University in St Louis, UC Irvine and the Association for Frontotemporal Dementia.

Andrea Slachevsky is supported by ANID / Fondap/ 15150012, ANID / Fondecyt/ 1231839, ANID/FONDEF/ ID22I10251; Alzheimer Association (ALZ‐RWD‐26‐1466627) and National Institute On Aging of the National Institutes of Health (R01AG075775, R01AG083799, 2P01AG019724); and the Multi‐partner Consortium to Expand Dementia Research in Latin America (ReDLat), which is supported by the Fogarty International Center and the National Institutes of Health, the National Institute on Aging (R01AG057234, R01AG075775, R01AG21051, and CARDS − NIH), Alzheimer's Association (SG‐20‐725707), Rainwater Charitable Foundation's Tau Consortium, the Bluefield Project to Cure Frontotemporal Dementia, and the Global Brain Health Institute.

Juliana N. Souza‐Talarico received grants and support for attending meetings and/or travel from the Alzheimer's Association.

Ezequiel I. Surace received support from the Alzheimer's Association for attending the AAIC Satellite Symposium Lima, Peru 2025.

Leonel T. Takada received grants from NIH (RedLat) and Alzheimer's Association (DIAN); support for attending meetings and/or travel from United Medical and participated on a Data Safety Monitoring Board or Advisory Board of Denali.

Eduardo R. Zimmer received grants from Alzheimer's Association, National Academy of Neurosphycology, CNPq, CAPES, FAPERGS, Michael J Fox, Serrapilheira, and Ciência Pioneira; consulting fees from Nintx, Novo Nordisk, Biogen, and Magdalena Biosciences; payment or honoraria for lectures, presentations, speakers bureaus, manuscript writing or Educational events from Novo Nordisk and Biogen; support for attending meeting and/or travel from Alzheimer's Association, CNPq, Capes, Novo Nordisk, and Biogen; and is Co‐founder and minority of MASIMA.

Simin Mahinrad is a full‐time employee of the Alzheimer's Association. As a full‐time employee of the Alzheimer's Association, all her travel for attending meetings and/or conferences is covered by her employer.

Heather M. Snyder is a full‐time employee of the Alzheimer's Association. She received grants from NIA and CDC. As a full‐time employee of the Alzheimer's Association, all her travel for attending meetings and/or conferences is covered by her employer. She participated in a Data Safety Monitoring Board or Advisory Board of NIA and NINDS‐funded initiatives including DISCOVERY AD and Microbiome AD/ADRD studies. She served as past board member of Health Research Alliance (unpaid); Research Committee of American Heart Association (unpaid); Liaison of Brain Health Council at American Heart Association (unpaid); Women's Brain Health Committee, AARP (unpaid); chair of CDMRP, DoD Alzheimer's and Related Disorders Committee (unpaid) and XPrize Judge (unpaid). Her Spouse works for Abbott in an unrelated area.

Maria C. Carrillo is a full‐time employee of the Alzheimer's Association. She has a daughter in the neuroscience program at USC. She reports receiving grants from NIA and CDC. As a full‐time employee of the Alzheimer's Association, all her travel for attending meetings and/or conferences is covered by her employer. She participated in a Data Safety Monitoring Board or Advisory Board of NIA and NINDS‐funded initiatives, including ADSP. She served on the board of GHR Foundation, and Research Committee at the American Heart Association (unpaid).

The remaining authors have nothing to disclose. Author disclosures are available in the Supporting information.

## Supporting information



Supporting Information
